# Can Exercise Make You Smarter, Happier, and Have More Neurons? A Hormetic Perspective

**DOI:** 10.3389/fnins.2016.00093

**Published:** 2016-03-14

**Authors:** Simona Gradari, Anna Pallé, Kerry R. McGreevy, Ángela Fontán-Lozano, José L. Trejo

**Affiliations:** Laboratory of Adult Neurogenesis, Department of Molecular, Cellular and Developmental Neurobiology, Cajal Institute, Consejo Superior de Investigaciones CientíficasMadrid, Spain

**Keywords:** exercise, adult hippocampal neurogenesis, biphasic dose-response, hormesis, molecular mechanisms, cognition, mood

## Abstract

Exercise can make you smarter, happier and have more neurons depending on the dose (intensity) of the training program. It is well recognized that exercise protocols induce both positive and negative effects depending on the intensity of the exercise, among other key factors, a process described as a hormetic-like biphasic dose-response. However, no evidences have been reported till very recently about the biphasic response of some of the potential mediators of the exercise-induced actions. This hypothesis and theory will focus on the adult hippocampal neurogenesis (AHN) as a putative physical substrate for hormesis responses to exercise in the context of exercise-induced actions on cognition and mood, and on the molecular pathways which might potentially be mediating these actions.

## Introduction

Physical activity induces pleiotropic effects for the whole organism including brain (Dishman et al., 2006). The effects of physical exercise can be described by means of a hormetic (biphasic) dose-response curve both on cognition and mood (Mattson, 2012a), and many of these effects have been closely related to the adult hippocampal neurogenesis (AHN) in the last decade (Kempermann, 2011). AHN is a phenomenon consisting of the formation of new neurons during adult life, and certainly these new neurons are highly responsive to exercise. AHN has also been related, independently of their response to exercise, to some hippocampus-dependent behaviors and mood (Kempermann, 2011).

Taking this knowledge into consideration, we have reviewed first the literature related to the hormetic profile effects of both the exercise and its molecular mediators on the brain. Following these evidences, we have posed and discussed the hypothesis whether AHN responses display a biphasic/hormetic dose-response to exercise and whether some putative mechanisms underlying this response profile may be detected in the literature.

The answer to this question is relevant for our knowledge about the neurobiology of exercise, but nevertheless, also to raise a property of AHN (hormesis), that can easily influence and improve our attempts to manipulate adult neurogenesis beyond exercise (drugs or other interventions).

## Exercise and brain

Physical activity is an essential component of everyday life. Searching for food or sexual partners, chasing preys or running away from predators, seasonal migrations, and almost any regular activity related to maintaining life is usually mediated by some kind of physical activity. These life-sustaining activities involving a physical component are intimately associated to cognitive activities. The processing of information about the surrounding environment where the organism is living and moving, learning, and storing such information, and its application for decision making are cognitive processes naturally occuring at the same time the organisms are moving.

Furthermore, the relationship between physical activity and brain information processing is more relevant than a mere correlational event. The way the brain processes information changes along with the level of physical activity of the organism (Foster, 2015), but even more important, physical activity effectively changes the brain, both at a morphological and functional levels. This adaptive brain plasticity responds to the different levels of information processing demand in the way of adjusting neural resources available to process that information level (Chen and Tonegawa, 1997). These neural resources and the available changes range from neural cell metabolism and gene expression, cell and processes size, neuronal dendrite and synapse number, and blood-brain barrier properties, to synaptic plasticity. While all these changes involve existing cells, neural resources also include a form of metaplasticity (named adult neurogenesis): the capability to recruit, in some specific areas of the adult brain, more neurons which show cellular and synaptic plasticity abilities (Garcia-Segura, 2009).

In this scenario, it is not surprising that physical exercise training has been largely reported as increasing synaptic plasticity, neural cells metabolism and blood supply in an active brain area-specific way, and increasing neural processing abilities, even in humans (in adults, children, and adolescents). Besides, in human beings a specific capability for endurance running, compared to the other primates has been postulated (Mattson, 2012b). Comparisons between animal and human studies are, therefore, difficult. However, common mechanisms underlie the aspects discussed in the present work, as for example, the inflection point for the effects of different intensity levels of exercise around the anaerobic threshold (lactate threshold) used in both laboratory rodent and human studies (see below). An increasing body of literature has long demonstrated benefits of exercise for cognition in humans (reviewed by Hillman et al., 2008). In experiments with laboratory rodents, it has been long demonstrated that exercise increases cognitive performance in almost any brain area, especially at hippocampus-dependent tasks, by means of synaptic plasticity and neurogenesis increments. It increases complexity of neuronal dendrites and synapse numbers. Regular exercise induces neuroprotection in all brain areas analyzed; it improves different parameters related to neurodegenerative diseases (age of onset, progression, and severity of symptoms) both in animal models and in humans. Finally, exercise has also been reported as a useful tool to recover the symptomatology of some neurodegenerative diseases and from different brain damages and insults (Dishman et al., 2006; Mattson, 2015). From a mechanistic point of view, exercise is able to increase blood flow (Lucas et al., 2015) and oxygen consumption (Rooks et al., 2010), modulate the growth factor signaling cascade (especially IGF1 Llorens-Martin et al., 2009, BDNF Gomez-Pinilla and Hillman, 2013, and VEGF During and Cao, 2006), and increase neurotransmitter availability and function (especially dopamine, glutamate, norepinephrine, and serotonin Meeusen, 2012).

Nevertheless, exercise displays inseparable negative effects. These stressors range from thermal, metabolic, hypoxic, and oxidative, to mechanical stress (reviewed by Peake et al., 2015). These effects have been reported both in animal and human studies. The higher capacity for endurance running showed by human beings (Mattson, 2012b) would just be one stage of the hormetic response by which a low dose of a stressful stimulus activates an adaptive response that increases the resistance to a moderate-to-severe level of stress (Calabrese et al., 2007). However, a number of different approaches have demonstrated inverted-U performance curves on cognition after exercise in humans (Tomporowski, 2003). The relationship is not simple because it clearly depends on the duration of the bouts (typically intense but brief, leading to a rapid recovery of the individual's capacity) and on the individual's fitness level previous to the study, a factor under the lack of clear effects of brief bouts of intense exercise (Tomporowski, 2003), and on the motivation to participate in both acute bouts of exercise or long endurance training regimes, a factor that surely distorts any conclusion about relationships between exercise and cognitive performance in humans. On the other side, steady-state aerobic exercise causes impaired information processing and cognition depending on the level of dehydration, in turn depending on the duration of the exercise (Cian et al., 2001). As the most outstanding factor, exercise increases oxidative stress, as expected as it consists of a physical increased activity of oxygen-consuming organisms and this effect is intimately associated to variations in energy metabolism (Coyle, 2000). This aspect of exercise programs can well be underlying the negative effects of strenuous physical activity (Nieman et al., 1990; Lee et al., 1995; Andersen et al., 2013). As it is well-known that exercise displays this kind of hormetic-like non-monotonic response curve for most outcomes analyzed (Radak et al., 2005), it is relevant to take into account that this biphasic response strongly depends on oxidative stress, oxygen consumption and mitochondrial metabolism. All these evidences are related to the effects on the whole body, mainly muscle and cardiovascular systems. As for the brain, it is important to take into account that exercise induces increased generation of ROS but also antioxidant enzymes and redox signaling in the body (Powers and Jackson, 2008; Radak et al., 2013), and oxidative stress can be induced into the brain by circulating factors (Calabrese et al., 2010). As general positive effects of exercise on brain's oxidative stress has long been recognized due to an increased activity of antioxidant enzymes (reviewed by Radak et al., 2014), it has been suggested that a complex regulation and balance between exercise-induced generation of sistemic ROS-related factors and brain antioxidant enzymes are mediating the effects of exercise on the brain in a hormetic, bell-shaped curve, where high levels of ROS cause oxidative damage, while moderate amounts would induce an adaptive response to oxidative challenge (Radak et al., 2014).

Moreover intense, above lactate threshold level exercise induces brain mitochondrial dysfunction and decreased BDNF levels in mice (Aguiar et al., 2008), increased activity of hypothalamic paraventricular nucleus and concomitant increase in CRH expression (Timofeeva et al., 2003), suggesting a direct activation of stress responses, while others have found only positive effects of intense exercise in aged mice (Lezi et al., 2014).

A word of caution is worth to be taking into account at this time. For the present work, we have considered that the broad range of parameters related to exercise are the same for humans, rats and mice, although subtle differences exist, not only between species, but also between mouse strains, gender and even inter-individual variability. Therefore, aerobic capacity, lactate and anaerobic thresholds and critical speed show similar features and the same curve profiles for human, rats and mice (Billat et al., 2005), as well as similar characteristics of maximal lactate steady state (MLSS; Ferreira et al., 2007), although noticeable differences do exist, such as a different sensitivity of MLSS for mice to endurance training compared to humans and rats (Beneke et al., 2000; Gobatto et al., 2001; Ferreira et al., 2007). All these works point to a very similar physiology of exercise and relevant parameters in laboratory rodents and humans, making the conclusions of this work relevant not only to know the specific features of rodent physiology but also to use animal models to study preclinical interventions. The most relevant distinction might be the subtle differences in the profile of aerobic–anaerobic transition between mice and both rats and humans. These differences are not critical for the topics discussed in the present work.

In this work, hormesis is considered the dual response of an individual's behavior, an organ or a cell's physiology or any other analyzed parameter in response to the graded intensity of a specific treatment/intervention (Mattson, 2008). In its most usual form, hormesis represents a dose-response curve with a biphasic profile: beneficial effects at a low dose/intensity and detrimental (inverted-J curve) or no effects (inverted-U curve) at a higher dose/intensity. Therefore, hormesis refers not only to negative effects at high doses, but also to a lack of moderate dose-induced positive effects at higher doses. As for exercise, hormesis adopts the features of pre-conditioning/adaptation to a mild, intermittent stress when coping with further higher stressors. This way, the hormesis curve is an adequate description of the effects of exercise (Radak et al., 2008). Therefore, it is relevant to consider the hormetic responses to physical exercise, focused on brain effects.

## Biphasic responses to exercise on cognition and mood

It is not the aim of the present work to determine which are the specific characteristics that are optimal to obtain beneficial or detrimental effects with an exercise training protocol, both because the comparison between different works have been demonstrated very difficult in a number of meta-analysis, due to the inability to equate exercise intensity levels between studies, and because it is known only scarcely the many different parameters of the exercise regime influencing the exercise outcomes. What is most relevant for this work is whether authors have found biphasic responses independently of the applied protocol. Anyway, examples of reports with some of the most representative forced exercise protocols in animal studies and its both positive and negative effects on stress biomarkers, neuroplasticity and behavior are included in Table 1. Protocols vary especially when forced exercise is considered, because voluntary running protocols use to depend largely on the animals' motivation to run.

**Table 1:** **Examples of positive, lack of positive, and negative effects of different forced training intensities on stress, behavior, and neurogenesis**.

**Animal model**	**Treadmill protocol**	**Stress biomarkers**	**Effects on cognition**	**Histological and molecular procedures**	**References**
Brain ischemia rat model: Adult Sprague-Dawley (SD) rats	- Sedentary (SED) group: 0 m/min - Low-intensity exercise (LI) group: 8 m/min - High-intensity exercise (HI) group: 20 m/min Duration in each group: 30 min/day for 14 days.	Serum corticosterone (CORT) Only HI group presented higher serum CORT concentration (levels around 200 μg/ml) than SED group.	Morris Water Maze (MWM) task LI but not HI group demonstrated a better spatial memory performance than SED group by spending more time in the target (platform) quadrant.	BDNF, Synapsin-I, PSD-95 Only the LI but not HI group presented increased levels of BDNF, Synapsin-I and PSD-95 in the contralesional hippocampus compared to the SED group.	Shih et al., 2013
Adult male Sprague-Dawley (SD) with severe cortical impact	- Sedentary (Control) group: 0 m/min - Low-intensity (LI) group: Progressive speed until reaching a maximum of 8 m/min from day 8 to day 14 (end of the protocol). - High-intensity (HI) group: Progressive speed until reaching 12 m/min from day 4 to the end of the running protocol. Duration: 30 min/day for 14 days.	No stress biomarkers were assessed	MWM task LI group had a shorter latency to locate the platform and a better performance in spatial memory compared to the control group. The HI exercise group showed a longer latency and a mild improvement in spatial memory compared to the control group.	BDNF LI group had increased levels of BDNF in the contralateral hippocampus respect de control group. p-CREB LI group had increased levels of p-CREB in the contralateral hippocampus respect de control group.	Shen et al., 2013
Adult rats	Treadmill with speed paradigm based on the lactate threshold (LT being around 20 m/min) - Sedentary control (CONT) group: Duration: 6 weeks - Stress free mild exercise (ME, < LT) group: Duration: 6 weeks. - Intense exercise (IE, >LT) group: Duration: 6 weeks.	Only IE causes general adaptive syndrome (GAS): hypercorticosteronemia, adrenal hypertrophy, thymic atrophy.	MWM task ME led to enhanced memory, but not learning, compared with CONT. IE produced no changes in either learning capacities, probably due to GAS.	Adult Hippocampal Neurogenesis (AHN) 2 weeks of training with stress-free mild exercise (ME), but not intense exercise (IE), comprising exercise stress, promotes adult hippocampal neurogenesis.	Inoue et al., 2015a
Adult male Wistar rats	Treadmill with speed paradigm based on the lactate threshold - Sedentary control (CONT) group: 0 m/min - Supra-lactate threshold (Middle speed) group: 25 m/min. - Sub-lactate-threshold (Low speed) group: 15 m/min. Duration: 30 min/day for 2 weeks.	Serum ACTH levels Significant increases in plasma ACTH were observed during supra-LT running.	No behavioral tasks were performed	cFos induction Only supra-LT running significantly increased c-Fos induction in various hypothalamic regions.	Soya et al., 2007
Male albino Sprague-Dawley rats (4–6 weeks old)	For 4 weeks: intensity of 70% of maximal oxygen consumption, for 1 h/day, 5 day/week.	No stress biomarkers were assessed	One-trial step-through passive avoidance test: ↑ learning and memory.	No histological or molecular procedures were performed	Chen et al., 2008
Adult Wistar Rats	Treadmill with speed paradigm based on the lactate threshold - Sedentary control (CONT) group: 0 m/min	Plasma CORT Only IE had the higher CORT concentration than CONT group.	No behavioral tasks were performed	AHN ME was better suited to improve AHN, especially in regards to the survival and maturation of newborn neurons.	Inoue et al., 2015a
- Mild-exercise (ME, < LT) group: 15 m/min, 60 min/day. - Intense-exercise (IE, >LT) group: 40 m/min, 60 min/day. Duration: 6 weeks in total including the habituation period. Running took place during the dark phase (19:00 and 22:00).			DNA microarray - ME-influenced genes were principally related to lipid metabolism, protein synthesis and inflammatory response, which are recognized as associated with AHN - IE-influenced genes linked to an excessive inflammatory immune response, known to be negative regulator of hippocampal neuroadaptation, were identified.		
Sprague-Dawley rats (5-weeks-old)	Treadmill: initial speed of 9 m/min for 20–60 min per day, 5 days per week for the first week, followed by 60 min/day at the same speed, 5 days/week. Increasing speed about 3 m/min per week reaching 16 m/min at the end of the training period. Running Wheel: singly placed in cages.	No stress biomarkers were assessed	Fear conditioning: No changes in the acquisition of fear-evoked conditional responses and ↑ context-conditioned freezing responses in treadmill and running wheel. Only treadmill improved the cue-conditioned performance.	No histological or molecular procedures were performed	Lin et al., 2012
Male juvenile Sprague-Dawley rats (5 weeks old)	For 1 week: 30 min/day. Three groups: - low intensity (LI) group: ran at 5 m/min for the first 5 min, 8 m/min for the next 5 min and 11 m/min for the remaining 20 min; - moderate intensity (MI) group ran at 8 m/min for the first 5 min, 11 m/min for the next 5 min and 14 m/min for the remaining 20 min; - high intensity (HI) group ran at 8 m/min for the first 5 min, 11 m/min for the next 5 min and 22 m/min for the remaining 20 min.	No stress biomarkers were assessed	No behavioral tasks perfomed	AHN ↑↑↑↑ BrdU^+^ and BrdU^+^/NeuN^+^ cells in the LI group; ↑↑↑ BrdU^+^ and BrdU^+^/NeuN^+^ cells in the MI group; ↑↑ BrdU^+^ cells in HI group; ↑ BrdU^+^ and BrdU^+^/NeuN^+^ cells in the control group.	Lou et al., 2008
Male Sprague-Dawley rats (2 weeks of age): induction of autism-like with valproic acid injections.	30 min/day, five times a week for 4 weeks, starting postnatal day 28. Speed of 2 m/min for the first 5 min, at a speed of 5 m/min for the next 5 min, and then at a speed of 8 m/min for the last 20 min, with the 0° inclination.	No stress biomarkers were assessed	Open field and social interaction test: ↑ spatial learning memory in the autistic rats; Radial 8-arm maze test: ↑ working memory in the VPA-injected rats with exercise.	AHN ↑ number of BrdU^+^ cells	Seo et al., 2013
Male Wistar rats subjected to surgery	For 1 week: 1h/day, 5-10 m/min.	No stress biomarkers were assessed	Object displacement task: ↑ spatial learning; Object substitution task: ↑ object recognition learning.	No histological or molecular procedures were performed	Griffin et al., 2009
C57BL/J6 mice	- Controls (CON): 0 m/min - Regular Runners (RR): 10 m/min, at the same time of the day until 28 days - Irregular Duration Runners (IDR): 10 m/min. Same time of the day but variable duration. - Irregular time-of-day runners (ITR): 10 m/min. Same duration but at different time of day.	Serum CORT levels Day 4: No differences were found among runners. Day 29: RR group had significantly lower levels of serum CORT (110-150ng/ml at 10:00 am).	MWM task The RR group had a lower escape latency in the acquisition compared to the CON or IDR group. Regarding memory consolidation, RR spent more time in the target quadrant compared to the other three groups.	RR group presented higher levels of BrdU^+^ cells compared to the other groups.	Li et al., 2013
C57BL/J6 mice	Forced Walking Wheel System - Sedentary: 0m/min - Low impact runners (LIR): 10 m/min. 45 min/day. Duration: 10 weeks. - High impact runners (HIR): 21m/min, 45 min/day. Duration: 5 weeks.	No stress biomarkers were assessed	MWM task In the acquisition phase, HIR had longer escape latencies compared to LIR group and sedentary controls. Regarding memory consolidation performance, LIR crossed the platform quadrant more than HIR. Rotorod test 5 weeks of HIR led to significant improvement in rotorod test performance.	No histological or molecular procedures were performed	Kennard and Woodruff-Pak, 2012
Adult male C57BL/6 mice	For 2 weeks: 7 days/week, 40 min/day, speed 12 m/min.	No stress biomarkers were assessed	No behavioral tasks perfomed	AHN ↑ number of BrdU^+^ cell; ↑ density of spine of granule cells in the DG	Glasper et al., 2010
Adult male C57BL/6 mice	For 2 weeks: 5 days/week, 40 min/day, speed 12 m/min.	No stress biomarkers were assessed	No behavioral tasks perfomed	AHN No changes in the number of mature granule neurons; ↑ number of DCX^+^/CLR^−^ cells; ↑ number of (DCX^+^/CLR^+^)/Granule neurons; ↑ total DCX^+^/Granule neurons; ↑ total CLR^+^/Granule neurons	Llorens-Martín et al., 2006
Adult male C57BL/6J mice (5-weeks-old)	10 m/min, 20 min for the first day, with an increment of 10 min/day until reaching 60 min/day to fulfill the 70% of maximal oxygen consumption. The running duration was 60 min/day, and the running speed was increased gradually from 10 to 12 m/min. The speed was accelerated 1 m/min every 2 weeks.	No stress biomarkers were assessed	No behavioral tasks perfomed	AHN ↑ number of Nestin^+^ cells in the SGZ; ↑ number of Ki67^+^ cells; ↑ more DCX^+^ cells, with prominently developed dendrites; ↑ pCREB expression; ↑ BDNF expression	Nam et al., 2014
Male BALB/c mice (3-months old)	For 4 weeks: 10 m/min, for 20-60 min/day, 5 days/week.	No stress biomarkers were assessed	One-trial passive avoidance: ↑ retention latency. Multiple-trial passive avoidance:	No histological or molecular procedures were performed	Liu et al., 2008
			↑ just the retention phase of memory (not the acquisition).		
C57BL/6 male mice (19 months)	For 8 weeks, 5 days/week, 2 sessions/day, 5° incline. For the first week, each session consisted of a 10-min warm-up at 15 m/min followed by 30 min at 18 m/min. During the following 7 weeks, treadmill speed was progressively increased every week. Specifically, for weeks 2, 3, 4, 5, 6, 7, and 8 the treadmill speed was set to 21 m/min, 22 m/min, 23 m/min, 24 m/min, 25 m/min, 25 m/min, and 26 m/min, respectively.	No stress biomarkers were assessed	No behavioral tasks perfomed	No changes in DCX mRNA levels; ↑ VEGF mRNA; No changes in BDNF mRNA levels	Lezi et al., 2014

*In general, the highest intensities lead to a higher concentration of stress biomarkers, and either to a lower improvement, no improvement or negative effects (compared to sedentary controls) in behavioral performance and neurogenesis. Most authors designate the different intensities according to the velocity of running, based on the assumption that a correlation exists between running speed and lactate threshold (LT) although most of them do not measure lactate in their studies, so we have used the different running velocity as a classification criterion. LT, lactate threshold. References included in the Table and not in the text are: (Van Praag et al., 1999; Griesbach et al., 2004; Adlard et al., 2005; Bjørnebekk et al., 2005; Eadie et al., 2005; Redila and Christie, 2006; Kohl et al., 2007; Soya et al., 2007; Stranahan et al., 2007; Naylor et al., 2008; Leasure and Decker, 2009; Berchtold et al., 2010; Creer et al., 2010; Falls et al., 2010; Lafenêtre et al., 2010; Kennard and Woodruff-Pak, 2012; Li et al., 2013; Shen et al., 2013; Shih et al., 2013; Fischer et al., 2014; Inoue et al., 2015a,b; Radahmadi et al., 2015)*.

Physical exercise in human beings presents a biphasic dose-response curve for a huge number of cognition/mood parameters, depending on the intensity level of exercise program. Specifically, inverted U-shaped curves are found with an inflection point around the lactate or ventilatory thresholds. In a recent meta-analysis (Oliveira et al., 2015) on Feeling Scale (Hardy and Rejeski, 1989), it has been demonstrated that the intensity of the exercise is the main determinant to establish affective responses (no matter whether the exercise is self-selected or imposed). Beneficial effects of exercise in humans have been largely reported in a dose-response way (see Gomez-Pinilla and Hillman, 2013 for a recent review and Cotman and Berchtold, 2002; Dishman et al., 2006; Ang and Gomez-Pinilla, 2007; Mattson, 2012a,b). In a similar way, the subjective perception of the performance is decreased after an intense exercise (Grebot et al., 2003), and the stress associated to high intensity exercise has been reported to impair working and declarative memory (Taverniers et al., 2010).

In laboratory rodents, it is very well-known the high number of beneficial effects that a moderate exercise program can induce (Cotman and Berchtold, 2002; Kramer et al., 2006; Cotman et al., 2007). These beneficial effects include a variety of different tasks ranging from water maze (Ding et al., 2006), passive avoidance (Samorajski et al., 1985; Radak et al., 2006), contextual fear conditioning (Baruch et al., 2004; Burghardt et al., 2006) to radial arm maze (Schweitzer et al., 2006). On the contrary, it has been reported that high intensity exercise induces a variety of detrimental effects, including brain region-specific (amygdala - dorsal striatum becoming affected while hippocampus seem to be unaffected) and task-specific (differences found in some associative learning tasks-tone/shock conditioning-but not others-fear conditioning-and not in spatial reference and working memory tasks-water maze—) impairments in memory processing (Aguiar et al., 2010). Similar results have been found for passive avoidance tasks and contextual fear conditioning closely associated to brain oxidative stress (Rosa et al., 2007). Other authors have reported impairment of spatial-water maze-learning after high intensity exercise by using a different ergometer (treadmill running), although only at early acquisition phase (Blustein et al., 2006). High intensity levels of exercise can promote no improvements at all on cognition even after voluntary running and selective breeding for high levels of voluntary exercise (Rhodes et al., 2003). The deleterious effects of high intensity exercise can also rely on individual variability, depending on the basal performance level previous to training (Braszko et al., 2001).

To our knowledge, very few studies have reported beneficial effects of high intensity exercise on cognition. It is noteworthy to mention one work reporting an improved memory performance in both strenuous and over-training exercise programs in a passive avoidance test (Ogonovszky et al., 2005). Surprisingly, this work found an increase in the BDNF levels only in the group performing most intense exercise.

As for the biphasic dose-response to exercise in animal studies, a recent work has reported evidences of biphasic dose-response curves for exercise effects on cognition in laboratory mice. Memory retention in an object recognition task was significantly improved at low-moderate intensity exercise while high and very-high intensity exercise induced no and negative effects, respectively, on discrimination (García-Capdevila et al., 2009).

The features of inverted-U shaped dose-response curves (the maximum-hormetic-response, the width of hormetic zone, the No Observed (Adverse) Effect Level (NOAEL), the distance to NOAEL and the zero equivalent point (Calabrese, 2008) to exercise are quite relevant considering that they strongly depend on the animal's previous health, brain specific outcomes analyzed, and parameters of exercise programs including whether the exercise is voluntary or forced (Radák et al., 2001, and see below).

A very relevant aspect of the hormetic response to exercise is the biological meaning. As already stated, sedentary life has well-known detrimental effects on brain functioning, while exercise is one of the most outstanding conducts in order to maintain health as well as a healthy aging, both in animal and humans. It is plausible to postulate that exercise effects are increasingly positive within the range from sedentary life to moderate and high intensity exercise. This response to exercise would show a sigmoidal profile, due to a ceiling effect: beyond a given level of exercise intensity, training time, or frequency of training, no further positive effects would accumulate due to a maximum plastic capacity of our body and brain to change in response to activity and/or to a maximum ability to modify the body and brain's performance. The evidences reviewed above suggest that this is not the case. The response to exercise seems to fit better with a hormetic profile where increasing intensity, training time, or frequency of training cause the disappearance of the positive effects of low-to-moderate exercise. Why might this be so? As exposed above, exercise is a stress. The adaptive effects of exercise on both muscles, bones, immune system, cardiovascular system, and brain make the whole body healthier and resistant to further stress. But nevertheless, there is a threshold of intensity from where the exercise-induced stress leads to non-positive, even in some cases detrimental effects (an inverted-U or even an inverted-J hormetic curve) due to the canonical long-term actions of stress hormones in the whole body. Therefore, the inherent stress associated to physical exercise might be postulated under the hormetic profile of responses to exercise. Anyway, it will be below presented a second putative explanation for the biological evolutionary meaning of hormetic responses to exercise focused on AHN.

## Molecular mediators of exercise actions

A long list has been accounted with the factors responsible for the different effects of exercise on brain, both positive and negative. Among those most relevant, an activity-driven growth factors cascade including IGF1, BDNF, and VEGF (Cotman et al., 2007; Llorens-Martin et al., 2008; Pérez-Domper et al., 2013; Szuhany et al., 2015) has been postulated as responsible for most of the beneficial effects together with the anti-inflammatory actions (Silverman and Deuster, 2014), while the oxidative stress signaling has been pointed out as the most outstanding detrimental factor after exercise. Other factors with a clear, direct influence on the outcomes of exercise training protocols are diet and lifestyle (Gomez-Pinilla, 2008). It is far beyond the scope of this review to mention an extensive list of molecular mediators of exercise actions. A well-known consensus (Mattson et al., 2004) establishes physical-cognitive activity and dietary restriction as inducing a mild, metabolic stress on neural cells (through increased levels of intracellular calcium and reactive oxygen species). This pathway activates several transcription factors like CREB and NF-kB, controlling BDNF and anti-apoptotic gene (such as Bcl-2) expression. These factors drive cell survival, synaptic plasticity and neurogenesis processes.

In the present work, we aim to emphasize the increasing body of evidences showing that both positive and negative effects of exercise have usually been found mediated by the same factors in a hormetic-like biphasic dose-response. To cite just a few examples, many of the positive effects of exercise depend on the concomitant increases of BDNF (Marosi and Mattson, 2014) and IGF1 levels (Llorens-Martin et al., 2009), as well as calorie restriction interacts with the individual's activity level (Mattson, 2000; Dietrich and Horvath, 2012), while high levels of BDNF and IGF1 may induce negative effects on the brain (Gwag et al., 1995; Ramsden et al., 2003) and the energy intake is associated to the risk of developing neurodegenerative diseases in a biphasic dose-response. Furthermore, it is not casual that all these factors and lifestyles are directly related to energy balance.

While all the above-mentioned factors are directly involved in the effects of physical training on cognition and mood, in the last decade a growing literature has accumulated pointing to the adult neurogenesis to be closely related to these changes and under the direct action of those growth factors.

## Exercise actions and adult hippocampal neurogenesis

Adult neurogenesis is the production of new functional neurons in the adult brain (Kempermann, 2011). It is well recognized that physical activity influences the level of adult neurogenesis in hippocampus as well as learning recruits newborn neurons in both hippocampus and olfactory system, and environmental enrichment increase immature neurons survival (see classical reviews by Kempermann et al., 1999; Mattson, 2000; Olson et al., 2006, and an extensive, recent review in Kempermann, 2011). Some evidence also suggests a potential effect of exercise on the olfactory system (Chae et al., 2014) although conflicting evidence has also been reported (Brown et al., 2003). This kind of experience-driven plasticity has even been proposed as a necessary process to the fine tuning of brain functioning (Opendak and Gould, 2015). A number of different mechanisms has been demonstrated in close relationship to this exercise actions on adult neurogenesis, the most outstanding being IGF1 (Trejo et al., 2001, 2008; Llorens-Martin et al., 2009; LLorens-Martín et al., 2010; Glasper et al., 2010), BDNF (Bekinschtein et al., 2011; Rothman and Mattson, 2013; Vivar et al., 2013), VEGF (Fabel et al., 2003; During and Cao, 2006), and Wnt pathway (Chen and Do, 2012; Bayod et al., 2014), to cite just a few. Examples of reports with some of the most representative exercise protocols and its effects on cognition and AHN are included for both forced (in Table 1) and voluntary exercise (in Table 2).

**Table 2:** **Examples of voluntary exercise protocols (running wheel)**.

**Animal model**	**Running wheel protocol**	**Behavior**	**AHN and neuroplasticity**	**References**
Adult male Sprague-Dawley rats	For 2 weeks	No behavioral tasks perfomed	↑ cellular proliferation in the SGZ; ↑↑ number of Ki67^+^ cells in the SGZ; ↑ total length of granule cells dendrites; ↑ spine density	Eadie et al., 2005
Adult male Sprague-Dawley rats	For 2 weeks	No behavioral tasks perfomed	↑ cell proliferation; ↑ granule cells with single primary processes	Redila and Christie, 2006
Adult male Sprague-Dawley rats	For 2 months	No behavioral tasks perfomed	↑ spines; ↑ dendrite length; ↑ arborization complexity in the DG	Stranahan et al., 2007
Female Long-Evans rats	In social isolation condition for 10 days	No behavioral tasks perfomed	No changes in the number of BrdU^+^ cells	Leasure and Decker, 2009
Flinders sensitive line (FSL) rats (a genetic model of depression)	For 1 month	Forced Swim Test: ↓ time of immobility than sedentary control	4 days after Forced Swim Test: ↑ cell proliferation in SGZ	Bjørnebekk et al., 2005
Male Sprague-Dawley adult rats subjected to a lateral fluid percussion injury	From day 0 to day 6 post-injury	No behavioral tasks perfomed	↑ plasticity markers in the sham operates, but ↓ in the injured rats	Griesbach et al., 2004
Adult female C57BL/6 mice (3 months old)	For 2 to 4 months	Morris Water Maze (between day 30 and 49): with 2 trials/day, runners decrease path length and latency to the platform	↑ BrdU^+^ and BrdU^+^/NeuN^+^ cells	Van Praag et al., 1999
Adult female C57BL/6JRj mice (10 weeks old)	For 5 days	No behavioral tasks perfomed	↑ proliferation; No changes in the S-phase and total cell cycle length; ↓ G1 phase	Fischer et al., 2014
Adult male C57bl/6 mice (2 months of age)	For 3 weeks	Radial Arm Water Maze: ↑ cognitive performance	No histological or molecular procedures were performed	Berchtold et al., 2010
Adult male C57bl/6 mice (2 months of age)	For 2 weeks	Fear conditioning: ↓startle amplitude in the absence of the tone both before and after conditioning; No changes in shock sensitization of startle; ↑ cued conditioned fear.	No histological or molecular procedures were performed	Falls et al., 2010
Adult male C57BL/6 mice (3 months old)	For 23 km	Pattern separation: ↑ enhanced spatial touch-screen performance when stimuli were presented in close proximity in adult mice	↑ number and density of BrdU^+^ cells; No changes in number of BrdU^+^/NeuN^+^ cells	Creer et al., 2010
C57BL/6 mice subjected to 6-Gy irradiation at P9	Introduced to a running wheel at 9 weeks of age for 4 weeks	Open-field test: running alleviates irradiation-induced behavioral alterations.	Exercise after irradiation: ↑ number of BrdU^+^/NeuN^+^ cells; ↑number of GFAP^+^/Sox2^+^ cells; No changes in the number of DCX^+^ cells	Naylor et al., 2008
Female TgCRND8 mouse line (encondes a double mutant form of APP 695)	For 1 month	Morris Water Maze: ↑ performance on day 1 and day 2; No changes in the probe trial	No histological or molecular procedures were performed	Adlard et al., 2005
Female R6/2 mice (a transgenic model of Huntington's disease)	5 mice per cage with access to two running wheels for 4 weeks	No behavioral tasks perfomed	No changes in cell proliferation; No changes in the number of neural progenitor cells; No changes in the survival of newborn hippocampal neurons	Kohl et al., 2007
Female synRas (a mouse model with reduced neurogenesis) (2–3 months old)	For 12 days	Novel object recognition task: ↑ performance	↑ proliferation rate; ↑ density of DCX^+^ cells; ↑dendritic arborization of the immature neurons	Lafenêtre et al., 2010

*Voluntary running increases hippocampal neurogenesis and improves learning. References included in the Table and not in the text are: (Llorens-Martín et al., 2006; Chen et al., 2008; Liu et al., 2008; Glasper et al., 2010; Lin et al., 2012; Lezi et al., 2014; Nam et al., 2014)*.

## Biphasic responses of adult hippocampal neurogenesis

AHN has been reported displaying hormetic-like non-monotonic response curves after several treatments/interventions. One example of this biphasic response is driven by adrenal steroids, in turn one of the best known cases of biphasic responses in the brain (Joëls, 2006). A dual population of receptors mediates the mechanism with different affinities for the substrate and very different actions. At low concentrations, glucocorticoids induce supportive and survival actions on dentate granule neurons of the hippocampus by mean of the mineralocorticoid receptor, while at higher concentrations, a deleterious effect is achieved through the glucocorticoid receptor (Sapolsky et al., 1986; McEwen, 2012). A similar scenario takes place when considering the role of glucocorticoids and adult neurogenesis (Schoenfeld and Gould, 2013). While low levels of glucocorticoid receptor (GR) activation is maintained (as for example while living in an impoverished environment, or with a sedentary lifestyle), low levels of adult neurogenesis are observed (both cell proliferation and maturation), while an enriched environment, regular moderate exercise, or learning, generates a normal GR activation and consequently, basal levels of adult neurogenesis. On the other end, high GR activity driven by uncontrollable stress decreases dramatically the neurogenesis by affecting both precursor proliferation and immature neuron differentiation. This scenario adopts a well-known inverted U-shaped biphasic response (Saaltink and Vreugdenhil, 2014). The mechanisms of adrenal steroids-induced inhibition of cell proliferation in the dentate gyrus (Gould and Tanapat, 1999) are mediated by N-Methyl-D-aspartate receptor (Cameron et al., 1997).

Apart from this well-known example of hormetic response, a body of evidences has accumulated in recent years with further examples. Thus, neural stem cells treated with low levels of chemical, physical or pharmacological stimuli (otherwise high concentrations being toxic) have been shown to protect these cell precursors when the brain becomes affected by a neurodegenerative disease (reviewed in Wang, 2013). One recent work has demonstrated that the proneurogenic effect of calorie restriction is mediated by the receptor for the orexigenic hormone acyl-ghrelin and the neurogenic transcription factor Egr1 (Hornsby et al., 2016). This receptor is involved in the hormetic-like response of the adult neurogenesis to energy balance. In a similar way, the neurogenic effect of some ginsenosides has been shown with a biphasic dose- and time-dependent regulation (Liu et al., 2007), a property shared by a growing number of phytochemicals (reviewed by Mattson et al., 2007). Certainly, the list of biphasic responses of AHN to different compounds is enormous, including oxytocin (Leuner et al., 2012), bisphenol (Kim et al., 2011), allopregnanolone (Wang, 2014), lead toxic exposition (White et al., 2007), statins (Chen et al., 2003), and even fluoxetin (Guilloux et al., 2013). Nevertheless, both VEGF and TGF-β display a biphasic action on neurogenesis. High doses of VEGF downregulates endogenous VEGF receptors (increasing neuronal differentiation and decreasing progenitor proliferation). Low dose upregulates VEGF receptors (with no clear effect in proliferation or differentiation, Meng et al., 2006). Low doses of TGF-β promote neurogenesis while high doses induce apoptosis in autonomic gangliogenesis (Hagedorn et al., 2000).

Therefore, as a body of evidences has been accumulated about biphasic responses to exercise of a variety of brain parameters including cognition and mood, and about biphasic responses of AHN to a variety of factors, and AHN has been postulated as a necessary mediator of many of the effects of exercise, the next question is whether biphasic responses of AHN exist to exercise.

## Hypothesis and first evidences supporting a biphasic response of adult hippocampal neurogenesis to exercise

Taking into account the above indirect evidences and two direct evidences recently reported (see below), we propose the hypothesis that adult neurogenesis might be a physical substrate for hormetic responses to exercise on cognition and mood. This hypothesis must be tested by analyzing the response of a battery of parameters associated to the AHN to increasing levels of exercise intensities, with higher intensities being above lactate (anaerobic) threshold (Billat et al., 2005). We suggest that this hypothesis would fit to the cited literature, to the direct evidences mentioned below, and to future data testing this possibility, best than the sigmoidal dose-response reported up to date. However, the dose-response of AHN to exercise is very well-known (Holmes et al., 2004). This response was initially found to be monotonic, as raising the exercise intensity (exercise volume) from the basal level of sedentary control animals rapidly leads to increases in neurogenesis rate. Not so many evidences have been accumulated for biphasic responses when exercise intensity reaches strenuous or very high levels. In fact, to our knowledge only a few works have reported evidences in this direction. A work reports enhanced neurogenesis (BrdU/NeuN-double immunoreactivity, no stereological methods) and increases in BDNF, NMDAR1, and Flk-1 mRNA only after low intensity exercise, while high intensity exercise brought all parameters back to control, sedentary levels (Lou et al., 2008). A second group, which had previously demonstrated a ceiling effect in the exercise actions above lactate threshold (intense exercise) in spatial memory and in AHN in rats (Inoue et al., 2015a,b), have also reported recently a significant inverted U-shaped dose-response of the BrdU/DCX-positive cells in the adult dentate gyrus to increasing levels of exercise (sedentary animals, subLactate threshold, and supraLactate threshold exercise), being the number of newborn, immature neurons increased only in the subLactate threshold, mild exercise group, together with no increase in ACTH or CORT levels (Okamoto et al., 2015). Interestingly, this neurogenesis-promoting effect of sub-stress threshold (mild) exercise depends on glucocorticoid receptor activation, suggesting a facilitative, permissive role of GC receptors, and moderate glucocorticoid levels during mild exercise. These evidences, together with the above mentioned hormetic-like responses to GC actions of hippocampus, make glucocorticoid receptors to be suggested a very plausible mechanism mediating the hormetic, biphasic dose-response of adult neurogenesis to the different intensity levels of exercise. But GRs might not be alone in these responses.

Finally, other work reported that other forms of hormetic U-shape responses of AHN to exercise exists, as a lack of significant pro-proliferative effects induced by exercise has been reported over time (Kronenberg et al., 2006). Therefore, hormetic responses to exercise of adult neurogenesis can be observed not only as a consequence of increasing doses but also after long-term trainings.

It is worth to consider what can the biological meaning of this profile response to exercise be. As discussed above in Section Biphasic Responses to Exercise on Cognition and Mood, the inherent stress associated to exercise can induce a detrimental increment in stress hormone levels, counteracting the positive actions of exercise beyond a given intensity level in the whole body including brain. The sensitivity to these hormones can even be more relevant in adult hippocampus as the higher brain region involved in the control of stress hormone levels in the body, as well as when considering the specific sensitivity of AHN to stress hormones concentration. Besides, we suggest a second further explanation for this profile specifically for adult-born neurons. It is tempting to speculate that an increasingly high number of newborn neurons in the adult hippocampus is not necessarily endlessly positive but much on the contrary, a very high number of newborn neurons can be detrimental for the functioning of the tri-synaptic hippocampal circuit. A too high number of newborn, immature neurons with specific and unique electrophysiological, morphological and connectivity properties into a fully mature circuit with mature granule neurons showing different physiological properties might cause an altered functioning of the system, after a very high intensity exercise. The evidences presented in this work may well be interpreted under the light of the proposed hypothesis, and as the adaptation of the system in response to very high intensity exercise to avoid the newborn neurons to accumulate beyond a given number, thereby the inverted-U response curve.

## Mechanisms of biphasic responses of adult hippocampal neurogenesis to exercise

As a second part of our hypothesis and taking into account the mentioned literature, we also propose that the growth factors (IGF1 and BDNF among the main key factors) mediating actions of exercise on AHN, may be potential candidates to mediate this response curve. Apart from GRs, some other molecular mediators of exercise actions on AHN have also been described displaying a hormetic-like dose-response for distinct parameters on brain, other than neurogenesis, thereby being putative candidates for the mediation of biphasic responses of neurogenesis. IGF1 has been reported as one of these factors, as a few exercise bouts induce no changes or even decrease serum levels (Kraemer et al., 1995), while long-term training increases IGF1 levels above pretraining values (Eliakim et al., 2006). IGF1 is able to induce neuronal rescue at low doses while no or opposite effects can be elicited at higher doses both *in vivo* (Johnston et al., 1996) and *in vitro* (Florini et al., 1986). No clear evidences exist about the mechanisms underlying this biphasic effect of IGF1 on brain, but a plausible suggestion is the well-known biphasic effect of insulin-like growth factor binding proteins, which are modulating the availability of IGFs for the canonical IGF receptors. In the choroid plexus, IGFBP2 enhances IGF1 biological actions at low levels while decrease the actions at higher concentrations (Delhanty and Han, 1993). These biphasic actions of IGF1 are also found in other organs like kidney (Wang et al., 2012), or muscle (Florini et al., 1986).

In the same way, secretion of VEGF in retinal epithelial cells to regulate cell proliferation displays a hormetic-like curve to hydroxynonenal in response to oxidative stress (Vatsyayan et al., 2012).

Finally but nevertheless, reactive oxygen species (ROS) can probably be mediators for hormesis in the responses to exercise (Radak et al., 2005; Goto and Radák, 2010). As explained before, ROS are compounds necessarily generated as a consequence of physical activity, in a dose-dependent manner. Oxidative stress is a basic, crucial response to the “alteration of redox homeostasis” generated by exercise (Nikolaidis et al., 2012). Depending on their concentration, their actions can be beneficial (as regulatory mediators in signaling processes or maintaining redox homeostasis) or detrimental for all major cellular components (Dröge, 2002). The stimulatory effects of low doses of ROS after moderate, intermittent stress, and the negative effects of high ROS dose after higher intensity exercise display a typical U-shaped dose-response curve. This hormetic profile is a major feature of adaptive stress responses, the underlying concept being that intermittent, moderate challenges increase the resistance of many different organ systems to chronic or higher levels of stress, and exercise-generated ROS, although inducing lipids, proteins and DNA oxidative damage, also induce the activation of redox sensitive transcription factors and signaling pathways necessary for the adaptive response (reviewed in Radak et al., 2008; Mattson, 2014).

## Other factors influencing the effect of exercise

Of course, other factors are involved in the body's response to exercise: the ergometer used, voluntary versus forced exercise, and the test used to analyze the behavior after the exercise protocol, among others. Very contradictory results have been reported when voluntary versus forced exercise are compared. Some works have reported an increase in locomotion after a voluntary running wheel while forced treadmill induced a decrease, together with the same opposite effect of these protocols on the number of GABAA receptors in striatum (Dishman et al., 1996). In the same line, other work reported an anxiogenic effect after forced treadmill exercise while voluntary exercise induced no effect (Leasure and Jones, 2008). Both works suggest an anxiogenic effect of forced but not voluntary exercise. Interestingly, in the latter work the authors observed the anxiogenic effect independently of the distance the animals ran, that was the same in both the voluntary wheel and the treadmill, and being the critical factor the speed of the exercise, consistenty higher in voluntary runners; this way the exercise took shorter time for voluntary runners in a daily basis. In parallel, AHN was significantly increased in both groups, voluntary and forced, being this increase higher in forced runners. This discrepancy between behavior and neurogenesis outcomes is in contradiction to most of the literature. It is relevant to take into account that both works used the open field alone to report locomotion or anxiety-like behaviors, and no other test to support the conclusions (elevated plus maze, or novelty suppressed feeding to mention just a few). On the contrary, other work has reported an anxiogenic effect of only voluntary wheel running compared to forced treadmill, by measuring contextual fear conditioning together with an increase in c-fos neuronal activation in amygdala (Burghardt et al., 2006).

All these studies and many others also point out to the duration of the daily exercise protocol and the duration of the whole exercise program (weeks or months) as another very relevant parameter for the different outcomes observed. Interestingly, the larger effects have been reported for the shorter durations of exercise protocols, concomitantly with the social isolation status of the animals (Hatchard et al., 2014). Motivation to run of the laboratory rodents used in the different studies, and the type of behavioral task used to test the animal conduct after the distinct exercise regimes are clearly determinant, key factors. Different tasks may require very different levels of motivation, and more relevantly, can recruit the activation of very distinct neural circuits and brain areas (see a review in García-Capdevila et al., 2009); brain region specific adaptations to exercise has also been recently reviewed (Morgan et al., 2015). Finally, to adequately compare works of voluntary versus forced exercise, different intensity levels, or human versus animal studies, measurement of VO_2_ and VO_2*max*_ would greatly improve our ability to analyze different results and to distinguish confounding variables.

## Final remarks

As for many other factors/drugs/interventions with hormetic-like biphasic dose-response curves, hormetic responses to exercise can be highly relevant to take into account. For example, it is well-known that forced treadmill exercise can induce detrimental effects when performed after a deep brain surgery (Jun et al., 2012). In our hands, physical exercise on a treadmill after a surgical intervention of adult male mice modifies the response both in hippocampus-dependent behaviors and in adult neurogenesis, by which the exercise intensity level usually promoting positive changes in neuron morphology, anxiolytic, and procognitive effects, induces after surgery no changes or changes in the opposite direction (forthcoming results). These findings can be easily interpreted in the way that surgery is diminishing the maximum positive response to exercise, and shortening the hormetic zone of exercise biphasic effects (Calabrese, 2008, all of them typical hormetic parameters). In the same line, some authors have recently reported that intensity level of exercise must be shifted down after a previous stress has been experienced, to obtain the usual positive effects typically measured by a variety of physiological parameters (Kim et al., 2015).

## Conclusions

Some works have provided evidences that AHN might display hormetic-like biphasic dose-responses to exercise. A number of reports have also provided evidences that molecular mediators of exercise actions on neurogenesis, also respond with biphasic curves independently of their participation in the exercise-induced effects on AHN. Taking into account both groups of evidences, we propose the hypothesis that adult neurogenesis might be a physical substrate for hormetic responses to exercise on cognition and mood, and that the growth factors (IGF1 and BDNF among the main key factors) mediating actions of exercise on AHN, may be potential candidates to mediate this response curve, together with ROS.

## Author contributions

SG, AP, KM, ÁF contributed to analyze the literature, made the Tables, and revise and made corrections to the draft of the manuscript. JT conceived the scientific main question, analyze the literature, wrote the draft and the final version of the manuscript, and is the correspondence author.

### Conflict of interest statement

The authors declare that the research was conducted in the absence of any commercial or financial relationships that could be construed as a potential conflict of interest.
